# Caring for the Dying Child and Family

**Published:** 1988

**Authors:** Sally Curnick

**Affiliations:** Bristol Children's Hospital


### Caring for the Dying Child and the Family

**Sally Curnick**

Bristol Children's Hospital

1 in 700 children between the ages of 1–14yrs develop cancer each year. In the South West Region we expect up to 80 new patients each year, and 25–30 deaths. In the Bristol Paediatric Oncology Unit our experience is that it is better for most terminally ill children to be cared for at home. Both parents are usually available, and used to looking after their sick child, and the child is often happier in familiar surroundings, where life can be more normal.

Because management of children with cancer is highly specialised and it is rare, General Practitioners can have little expert knowledge of the subject. The initial symptoms are often vague, leading to a delay in diagnosis, and this can cause loss of confidence in the G.P. In addition the intensive treatment at the hospital results in a close association being formed with the hospital staff during a traumatic time. It is especially important therefore, that the family see the hospital and the G.P. working together to restore their confidence in the primary health care team for the future.

When families opt for home terminal care they still retain their links with the hospital. The consultant will nominate a clinical assistant, myself and the social worker already known to the family, to work with the G.P. and his team. Together we can offer twenty four hour support, pain and symptom control, counselling/guidance on how to communicate with the dying child and/or siblings, emphasis on living what is left of that life to the full, and eventually a 'good' death . The 'out of hour' calls do not fall exclusively on the G.P.; hospital staff often give families their own home telephone numbers for continued contact. Every effort is made to cater for the wishes and comfort of the child and his family within their environment. The G.P. needs the additional support of the hospital team, especially if he is single handedly acting as 24 hr carer, in addition to his normal duties.

Young children often require high doses of morphine to maintain pain control, and it is not unusual for us to prescribe between 25–100 mgs 4 hourly. The oral route is our first choice, but suppositories may also be used if available. If a central venous catheter is inserted, the parents and/or patient are taught how to flush the line with Hepsal, and subsequently how to give the required medication through the line, if this is the adopted route.

Honesty is regarded as the best policy from the time of diagnosis, so if the child is old enough, and with the parents agreement, the subject of death and dying might well be discussed. If parents need help to explain death to their children, I mention two books, which both use analogies to life and death using different forms of imagery*. Most children develop their own ideas on death from quite an early age, from experiences such as losing an aged relative or pet, or from school friends or television. They don't seem to fear death in the same way as many adults; one teenager was greatly relieved when he remembered he knew someone who had already died, as this 'auntie' had looked after him and his sister whilst their mother was in hospital for an operation, so she knew just how he liked his eggs. . . . . . . . .

Another 6 yr old recently asked if there was anything to do up in heaven? you know games and things? He seemed quite satisfied after discussing what possible games etc. there might be in heaven.

Many parents will never have experienced death in a close relation before and need to be encouraged to talk about their fears and fantasies of seeing their child dying, and the probable mode of death needs to be explained and discussed at length. A contact number should always be available, and an open invitation to return to the hospital at anytime should they so wish.

Financial assistance from either D.H.S.S. or local charities might well be needed to allay financial worries. Mother might well have given up work, and Father having time off. An attendance allowance can be applied for if the child has been ill for six months or longer. Friends and neighbours who all often want to help, can be very useful in organizing meals for the family; time is very precious and the sick child/siblings are demanding extra attention and energy, so to have meals organized is a wonderful luxury.

Life needs to be as normal as possible, which might include attending school or nursery part time, the visit of a domicillary school teacher and having friends in to play, otherwise the child can feel very insecure and become frustrated or demanding and aggressive. Stories and videos often become a normal pastime whilst undergoing treatment for cancer, and these are very useful for occupying some of the waking hours when concentration is at a minimum. Siblings feel more secure if their life is as routine as possible, it gives school children their peer support, and an escape route from their fears, anger and jealously over the sick child, and the attention he attracts. Siblings should be encouraged to 'help' if they want to and should not be excluded from the focus of attention. If they are old enough, it is advisable to give them a choice if they want to be present when the child dies, if they want to view the body afterwards, and/or attend the funeral.

As the child deteriorates and appetite diminishes, oral hygiene is important, not only to guard against infection but to encourage a fluid intake. It is also necessary to ensure a regular bowel movement, as constipation is so common whilst taking opiate analgesics and can be very uncomfortable. Sheepskin rugs and senco mattresses are available on loan for the prevention of pressure sores when the child becomes less mobile. Wheelchairs and buggies are also available on loan to enable mobility to be retained for as long as possible.

Night-time can seem very long and frightening, and to have a MacMillan Nurse or a Marie Curie Sitter stay with the family is very reassuring. This allows the family to sleep knowing that someone is awake and watching over their child, ready to wake them if they are needed.

After the death, time is often needed to be spent with the child's body, perhaps putting chosen clothes on, or just holding and caressing until the family, parents and siblings are ready to call the undertaker. Funeral arrangements might have already been thought about, or not, depending on the family. Some teenagers think very carefully about their death, and like to make a will and divide some of their personal things amongst their family and friends. They may also express a preference for either burial or cremation, and sometimes choosing favourite hymns for the funeral service.

Regular contact is most important immediately following the death, or else the family will feel very isolated from all the support they've had during the terminal illness. As with all previous care, bereavement care needs to be individually tailored to suit each family. An added pressure for the parents can be that the siblings grief cycle is often much shorter in comparison to theirs, the surviving children may resent the prolonged grief of their parents, making them, the surviving children, feel insignificant. The first anniversary of the childs death is an important milestone, and most parents appreciate an acknowledgement that their child is not forgotten. Likewise during the first year there are other important dates such as the date of diagnosis, and of course birthdays and Christmas.

The consultants offer each bereaved family an appointment, to be taken up when the family feels they would like it, to deal with unanswered questions on any aspect of medical care. The period of bereavement goes on long after this first year, many parents have said "you never get over the death of a child, but in time the pain of that loss becomes more bearable".

My role is that of Family Support Nurse, and my job is to counsel the family, collectively and individually as the need arises. I also act as a liaison between the professionals in hospital and in the community. I am a link between the family and hospital staff. In this role I am able to utilise my previous experience as a Registered Sick Childrens Nurse and Community Nursing Sister and my training in humanistic psychology. The objective of my role and that of the few others like it around the country, is to allow parents faced with caring for a dying child to make a real choice between hospital and home care, and to have the necessary professional support to permit that decision.

*Waterbugs and Dragonflies, by Doris Stickney, publishers Mowbray.

Letter to a dying child, by Elisabeth Kubler-Ross, available from The Bristol Cancer Help Centre, Grove House, Cornwallis Grove, Clifton, Bristol BS8.

